# Poly[[bis­[μ-1,4-bis­(3-pyridylmeth­yl)piperazine-κ^2^ *N*:*N*′]dichlorido­cadmium(II)] tetra­hydrate]

**DOI:** 10.1107/S1600536809027767

**Published:** 2009-07-18

**Authors:** Karyn M. Blake, Robert L. LaDuca

**Affiliations:** a Lyman Briggs College, Department of Chemistry, Michigan State University, East Lansing, MI 48825, USA

## Abstract

In the title compound, {[CdCl_2_(C_16_H_20_N_4_)_2_]·4H_2_O}_*n*_, octa­hedrally coordinated Cd^II^ ions, situated on crystallographic inversion centres, bearing *trans*-disposed chloride ligands, are linked into (4,4)-grid coordination polymer layers by tethering 1,4-bis­(3-pyridylmeth­yl)piperazine ligands. The layers are aligned parallel to the (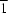
01) crystal planes and aggregate by means of O—H⋯N, O—H⋯O and O—H⋯Cl hydrogen-bonding mechanisms imparted by cyclic water mol­ecule tetra­mers.

## Related literature

For a cadmium succinate coordination polymer containing *N,N′*-bis­(4-pyridylmeth­yl)piperazine, see: Martin *et al.* (2009 ▶). For the preparation of *N,N′*-bis­(3-pyridylmeth­yl)piperazine, see: Pocic *et al.* (2005 ▶).
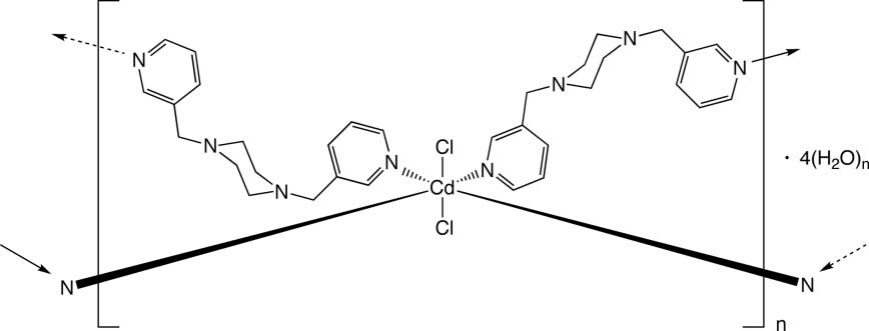


## Experimental

### 

#### Crystal data

[CdCl_2_(C_16_H_20_N_4_)_2_]·4H_2_O
*M* *_r_* = 792.08
Monoclinic, 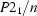

*a* = 10.3481 (2) Å
*b* = 13.9791 (2) Å
*c* = 12.7789 (2) Å
β = 92.4730 (10)°
*V* = 1846.84 (5) Å^3^
*Z* = 2
Mo *K*α radiation
μ = 0.78 mm^−1^
*T* = 173 K
0.38 × 0.35 × 0.19 mm

#### Data collection

Bruker APEXII diffractometer
Absorption correction: multi-scan (*SADABS*; Sheldrick, 1996 ▶) *T*_min_ = 0.753, *T*_max_ = 0.868
16443 measured reflections
3391 independent reflections
3060 reflections with *I* > 2σ(*I*)
*R*_int_ = 0.027

#### Refinement

*R*[*F* ^2^ > 2σ(*F* ^2^)] = 0.021
*wR*(*F* ^2^) = 0.055
*S* = 1.07
3391 reflections
226 parameters
6 restraints
H atoms treated by a mixture of independent and constrained refinement
Δρ_max_ = 0.42 e Å^−3^
Δρ_min_ = −0.23 e Å^−3^

### 

Data collection: *APEX2* (Bruker, 2006 ▶); cell refinement: *SAINT* (Bruker, 2006 ▶); data reduction: *SAINT*; program(s) used to solve structure: *SHELXS97* (Sheldrick, 2008 ▶); program(s) used to refine structure: *SHELXL97* (Sheldrick, 2008 ▶); molecular graphics: *CrystalMaker* (Palmer, 2007 ▶); software used to prepare material for publication: *SHELXL97*.

## Supplementary Material

Crystal structure: contains datablocks I, global. DOI: 10.1107/S1600536809027767/lh2863sup1.cif

Structure factors: contains datablocks I. DOI: 10.1107/S1600536809027767/lh2863Isup2.hkl

Additional supplementary materials:  crystallographic information; 3D view; checkCIF report

## Figures and Tables

**Table 1:** Hydrogen-bond geometry (Å, °)

*D*—H⋯*A*	*D*—H	H⋯*A*	*D*⋯*A*	*D*—H⋯*A*
O1*W*—H1*WA*⋯N2	0.874 (16)	2.084 (17)	2.950 (2)	171 (2)
O1*W*—H1*WB*⋯O2*W*^i^	0.884 (16)	2.007 (18)	2.838 (3)	156 (2)
O2*W*—H2*WA*⋯O1*W*	0.921 (17)	1.958 (19)	2.844 (3)	161 (3)
O2*W*—H2*WB*⋯Cl1^ii^	0.912 (17)	2.272 (18)	3.1607 (17)	165 (3)

Symmetry codes: (i) 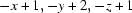; (ii) 
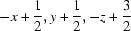
.

**Fig. 1.**
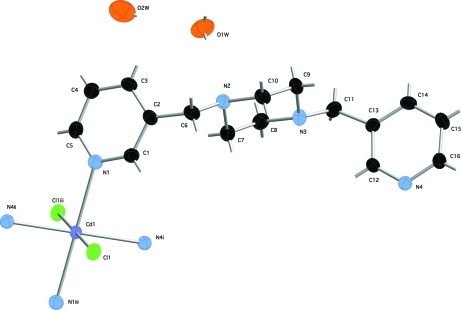
The expanded asymmetric unit of the title compound, showing 50% probability ellipsoids and atom numbering scheme. Hydrogen atom positions are shown as grey sticks. Color codes: violet Cd, green Cl, N blue, orange O, black C. Symmetry codes: (i) x+1/2, -y+3/2, z+1/2 (ii) -x-1/2, y-1/2, -z+1/2 (iii) -x, -y+1, -z+1.

**Fig. 2.**
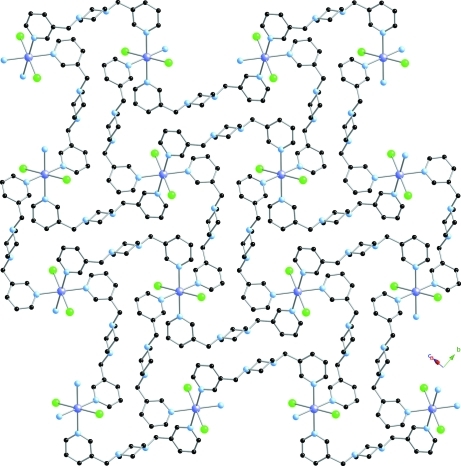
A view of the (4,4)-grid coordination polymer layer in the title compound.

**Fig. 3.**
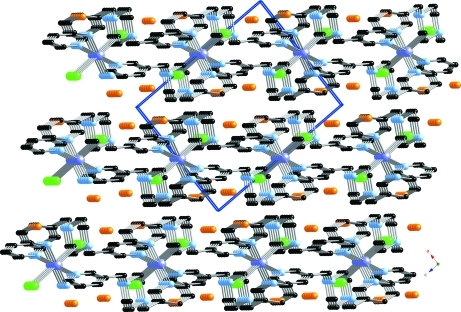
Stacking diagram of the title compound, viewed along the b crystal direction. Water molecule tetramers can be seen in the interlamellar regions.

## supplementary crystallographic information

### Comment

The title compound was prepared during an attempt to prepare a divalent cadmium coordination polymer containing both succinate and *N,N'*-di(3-pyridylmethyl)piperazine (3-bpmp) ligands. A cadmium succinate coordination polymer containing the isomeric *N,N'*-di(4-pyridylmethyl)piperazine (4-bpmp) ligand manifested the unique example of a 6^5^8 layered topology (Martin *et al.*, 2009).

The asymmetric unit of the title compound (Fig. 1) contains a Cd^II^ ion on the crystallographic inversion centre, one chloro ligand, one 3-bpmp ligand, and two water molecules of crystallization. Operation of the crystallographic symmetry generates an octahedral {CdCl_2_N_4_} coordination environment, with *trans* disposed chloro ligands and four N atom donors from pyridyl groups of four different 3-bpmp ligands.

Each Cd^II^ ion is linked to four others through the tethering 3-bpmp ligands to construct (4,4)-grid [CdCl_2_(3-bpmp)_2_]_n_ coordination polymer layers (Fig. 2) that are oriented parallel to the (1 0 1) crystal planes. The through-ligand Cd···Cd distances measure 10.658 (3) Å. The layers stack in an *AAA* pattern along the *a* crystal direction *via* hydrogen-bonding mechanisms provided by tetrameric water molecule aggregations (Fig. 3). Within a single coordination polymer layer, a water molecule (O1W) engages in O—H···N hydrogen-bonding with a piperazinyl N atom, and in turn with another water molecule of crystallization (O2W). Then, this second water molecule of crystallization provides O—H···Cl hydrogen-bonding to a chloro ligand. The water molecules of crystallization engage in mutual O—H···O hydrogen-bonding across the interlamellar regions to construct the cyclic water molecule tetramers.

### Experimental

Cadmium chloride dihydrate and succinic acid were obtained commercially. *N,N'*-bis(3-pyridylmethyl)piperazine was prepared *via* a published procedure (Pocic, *et al.*, 2005). A mixture of cadmium chloride dihydrate (81 mg, 0.37 mmol), succinic acid (44 mg, 0.37 mmol), *N,N'*-bis(3-pyridylmethyl)piperazine (99 mg, 0.37 mmol) and 10.0 g water (550 mmol) was placed into a 23 ml Teflon-lined Parr Acid Digestion bomb, which was then heated under autogenous pressure at 393 K for 48 h. The resulting yellowish solution was allowed to stand undisturbed at 293 K for 3 d. Large straw-colored crystals of the title compound were deposited.

### Refinement

All H atoms bound to C atoms were placed in calculated positions, with C—H = 0.95 Å and refined in riding mode with *U*_iso_ = 1.2*U*_eq_(C). The H atoms bound to water molecule O atoms were found in a difference Fourier map, restrained with O—H = 0.89 Å, and refined with *U*_iso_ = 1.2*U*_eq_(O).

### Crystal data

**Table:**

[CdCl_2_(C_16_H_20_N_4_)_2_]·4H_2_O	*F*(000) = 820
*M**_r_* = 792.08	*D*_x_ = 1.424 Mg m^−^^3^
Monoclinic, *P*2_1_/*n*	Mo *K*α radiation, λ = 0.71073 Å
Hall symbol: -P 2yn	Cell parameters from 16643 reflections
*a* = 10.3481 (2) Å	θ = 2.2–25.4°
*b* = 13.9791 (2) Å	µ = 0.78 mm^−^^1^
*c* = 12.7789 (2) Å	*T* = 173 K
β = 92.473 (1)°	Fragment, colourless
*V* = 1846.84 (5) Å^3^	0.38 × 0.35 × 0.19 mm
*Z* = 2	

### Data collection

**Table:**

Bruker APEXII diffractometer	3391 independent reflections
Radiation source: fine-focus sealed tube	3060 reflections with *I* > 2σ(*I*)
graphite	*R*_int_ = 0.027
ω and φ scans	θ_max_ = 25.4°, θ_min_ = 2.2°
Absorption correction: multi-scan (*SADABS*; Sheldrick, 1996)	*h* = −12→12
*T*_min_ = 0.753, *T*_max_ = 0.868	*k* = −15→16
16443 measured reflections	*l* = −15→15

### Refinement

**Table:**

Refinement on *F*^2^	Primary atom site location: structure-invariant direct methods
Least-squares matrix: full	Secondary atom site location: difference Fourier map
*R*[*F*^2^ > 2σ(*F*^2^)] = 0.021	Hydrogen site location: inferred from neighbouring sites
*wR*(*F*^2^) = 0.055	H atoms treated by a mixture of independent and constrained refinement
*S* = 1.07	*w* = 1/[σ^2^(*F*_o_^2^) + (0.0261*P*)^2^ + 0.7641*P*] where *P* = (*F*_o_^2^ + 2*F*_c_^2^)/3
3391 reflections	(Δ/σ)_max_ = 0.001
226 parameters	Δρ_max_ = 0.42 e Å^−^^3^
6 restraints	Δρ_min_ = −0.23 e Å^−^^3^

### Special details

**Table:**

Experimental. The fragment used in the single-crystal diffraction experiment was cleaved from a very large prismatic crystal using a scalpel.
Geometry. All e.s.d.'s (except the e.s.d. in the dihedral angle between two l.s. planes) are estimated using the full covariance matrix. The cell e.s.d.'s are taken into account individually in the estimation of e.s.d.'s in distances, angles and torsion angles; correlations between e.s.d.'s in cell parameters are only used when they are defined by crystal symmetry. An approximate (isotropic) treatment of cell e.s.d.'s is used for estimating e.s.d.'s involving l.s. planes.
Refinement. Refinement of *F*^2^ against ALL reflections. The weighted *R*-factor *wR* and goodness of fit *S* are based on *F*^2^, conventional *R*-factors *R* are based on *F*, with *F* set to zero for negative *F*^2^. The threshold expression of *F*^2^ > σ(*F*^2^) is used only for calculating *R*-factors(gt) *etc*. and is not relevant to the choice of reflections for refinement. *R*-factors based on *F*^2^ are statistically about twice as large as those based on *F*, and *R*- factors based on ALL data will be even larger.

### Fractional atomic coordinates and isotropic or equivalent isotropic displacement parameters (Å^2^)

**Table:**

	*x*	*y*	*z*	*U*_iso_*/*U*_eq_	
Cd1	0.0000	0.5000	0.5000	0.01972 (7)	
Cl1	−0.00576 (4)	0.56521 (3)	0.69090 (3)	0.02753 (11)	
O1W	0.31111 (17)	1.01315 (12)	0.44201 (15)	0.0498 (4)	
H1WA	0.267 (2)	0.9840 (17)	0.3915 (18)	0.060*	
H1WB	0.375 (2)	1.0362 (19)	0.4063 (19)	0.060*	
O2W	0.4410 (2)	0.92673 (13)	0.61847 (14)	0.0623 (5)	
H2WA	0.385 (3)	0.958 (2)	0.5722 (19)	0.075*	
H2WB	0.443 (3)	0.9689 (18)	0.6730 (17)	0.075*	
N1	0.16425 (14)	0.61714 (10)	0.46966 (11)	0.0256 (3)	
N2	0.14429 (14)	0.90557 (10)	0.29043 (11)	0.0242 (3)	
N3	−0.05153 (14)	1.05084 (11)	0.26799 (11)	0.0267 (3)	
N4	−0.35028 (14)	1.12026 (10)	0.05522 (11)	0.0235 (3)	
C1	0.14831 (17)	0.67064 (12)	0.38328 (14)	0.0254 (4)	
H1	0.0828	0.6526	0.3328	0.031*	
C2	0.22184 (17)	0.75090 (12)	0.36317 (14)	0.0246 (4)	
C3	0.31970 (18)	0.77468 (13)	0.43639 (15)	0.0301 (4)	
H3	0.3738	0.8283	0.4251	0.036*	
C4	0.33772 (19)	0.71988 (13)	0.52574 (16)	0.0336 (4)	
H4	0.4041	0.7354	0.5766	0.040*	
C5	0.25809 (18)	0.64246 (13)	0.53994 (15)	0.0308 (4)	
H5	0.2701	0.6055	0.6020	0.037*	
C6	0.19428 (18)	0.80989 (13)	0.26599 (14)	0.0281 (4)	
H6A	0.2748	0.8168	0.2276	0.034*	
H6B	0.1301	0.7760	0.2197	0.034*	
C7	0.02170 (18)	0.90049 (13)	0.34449 (15)	0.0290 (4)	
H7A	0.0356	0.8657	0.4115	0.035*	
H7B	−0.0430	0.8647	0.3006	0.035*	
C8	−0.0291 (2)	0.99965 (13)	0.36577 (16)	0.0309 (4)	
H8A	−0.1109	0.9950	0.4028	0.037*	
H8B	0.0344	1.0350	0.4112	0.037*	
C9	0.06858 (18)	1.05772 (13)	0.21285 (15)	0.0307 (4)	
H9A	0.1326	1.0953	0.2555	0.037*	
H9B	0.0521	1.0916	0.1456	0.037*	
C10	0.12320 (18)	0.95935 (14)	0.19196 (14)	0.0297 (4)	
H10A	0.0624	0.9237	0.1444	0.036*	
H10B	0.2062	0.9659	0.1569	0.036*	
C11	−0.11128 (18)	1.14457 (13)	0.28181 (15)	0.0312 (4)	
H11A	−0.0434	1.1930	0.2976	0.037*	
H11B	−0.1691	1.1423	0.3415	0.037*	
C12	−0.27629 (17)	1.10601 (12)	0.14168 (14)	0.0247 (4)	
H12	−0.2843	1.0468	0.1772	0.030*	
C13	−0.18808 (16)	1.17228 (12)	0.18317 (14)	0.0243 (4)	
C14	−0.17827 (18)	1.25856 (13)	0.13158 (15)	0.0298 (4)	
H14	−0.1203	1.3064	0.1578	0.036*	
C15	−0.25411 (18)	1.27477 (13)	0.04085 (15)	0.0311 (4)	
H15	−0.2482	1.3335	0.0040	0.037*	
C16	−0.33791 (17)	1.20436 (12)	0.00536 (14)	0.0261 (4)	
H16	−0.3892	1.2156	−0.0569	0.031*	

### Atomic displacement parameters (Å^2^)

**Table:**

	*U*^11^	*U*^22^	*U*^33^	*U*^12^	*U*^13^	*U*^23^
Cd1	0.02129 (11)	0.01716 (10)	0.02035 (10)	0.00052 (6)	−0.00332 (7)	0.00085 (6)
Cl1	0.0316 (2)	0.0285 (2)	0.0221 (2)	0.00443 (18)	−0.00335 (17)	−0.00290 (17)
O1W	0.0434 (10)	0.0459 (10)	0.0592 (11)	0.0018 (7)	−0.0069 (8)	−0.0213 (8)
O2W	0.0815 (14)	0.0578 (11)	0.0473 (10)	−0.0181 (10)	−0.0018 (9)	−0.0145 (8)
N1	0.0276 (8)	0.0227 (8)	0.0264 (8)	−0.0006 (6)	−0.0017 (6)	−0.0003 (6)
N2	0.0242 (7)	0.0247 (8)	0.0240 (7)	−0.0006 (6)	0.0033 (6)	0.0054 (6)
N3	0.0272 (8)	0.0269 (8)	0.0258 (8)	0.0029 (6)	−0.0011 (6)	0.0039 (6)
N4	0.0234 (7)	0.0221 (8)	0.0247 (7)	−0.0007 (6)	−0.0030 (6)	−0.0004 (6)
C1	0.0250 (9)	0.0246 (9)	0.0266 (9)	−0.0002 (7)	0.0008 (7)	−0.0025 (7)
C2	0.0247 (9)	0.0209 (9)	0.0288 (9)	0.0033 (7)	0.0070 (7)	−0.0010 (7)
C3	0.0267 (9)	0.0208 (9)	0.0426 (11)	−0.0027 (7)	0.0014 (8)	−0.0005 (8)
C4	0.0301 (10)	0.0288 (10)	0.0410 (11)	−0.0017 (8)	−0.0089 (9)	−0.0015 (8)
C5	0.0326 (10)	0.0276 (10)	0.0317 (10)	0.0009 (8)	−0.0044 (8)	0.0027 (8)
C6	0.0310 (10)	0.0261 (10)	0.0275 (9)	−0.0018 (8)	0.0059 (8)	0.0015 (7)
C7	0.0278 (9)	0.0306 (10)	0.0291 (10)	−0.0011 (8)	0.0055 (8)	0.0082 (8)
C8	0.0288 (10)	0.0368 (11)	0.0274 (10)	0.0037 (8)	0.0052 (8)	0.0048 (8)
C9	0.0308 (10)	0.0305 (10)	0.0308 (10)	−0.0018 (8)	0.0000 (8)	0.0093 (8)
C10	0.0300 (10)	0.0325 (10)	0.0269 (9)	−0.0008 (8)	0.0046 (8)	0.0074 (8)
C11	0.0312 (10)	0.0292 (10)	0.0324 (10)	0.0011 (8)	−0.0085 (8)	−0.0051 (8)
C12	0.0267 (9)	0.0190 (9)	0.0283 (9)	0.0002 (7)	−0.0011 (7)	0.0015 (7)
C13	0.0215 (9)	0.0230 (9)	0.0282 (9)	0.0011 (7)	−0.0016 (7)	−0.0033 (7)
C14	0.0272 (9)	0.0223 (9)	0.0396 (11)	−0.0049 (7)	−0.0021 (8)	−0.0041 (8)
C15	0.0370 (11)	0.0212 (9)	0.0349 (10)	−0.0023 (8)	0.0002 (8)	0.0047 (8)
C16	0.0293 (9)	0.0239 (9)	0.0251 (9)	0.0016 (7)	−0.0010 (7)	0.0021 (7)

### Geometric parameters (Å, °)

**Table:**

Cd1—N4^i^	2.3728 (14)	C4—C5	1.377 (3)
Cd1—N4^ii^	2.3728 (14)	C4—H4	0.9500
Cd1—N1	2.4036 (15)	C5—H5	0.9500
Cd1—N1^iii^	2.4036 (15)	C6—H6A	0.9900
Cd1—Cl1	2.6074 (4)	C6—H6B	0.9900
Cd1—Cl1^iii^	2.6074 (4)	C7—C8	1.511 (3)
O1W—H1WA	0.874 (16)	C7—H7A	0.9900
O1W—H1WB	0.884 (16)	C7—H7B	0.9900
O2W—H2WA	0.921 (17)	C8—H8A	0.9900
O2W—H2WB	0.912 (17)	C8—H8B	0.9900
N1—C1	1.338 (2)	C9—C10	1.515 (3)
N1—C5	1.342 (2)	C9—H9A	0.9900
N2—C7	1.472 (2)	C9—H9B	0.9900
N2—C6	1.472 (2)	C10—H10A	0.9900
N2—C10	1.474 (2)	C10—H10B	0.9900
N3—C8	1.450 (2)	C11—C13	1.511 (2)
N3—C9	1.458 (2)	C11—H11A	0.9900
N3—C11	1.463 (2)	C11—H11B	0.9900
N4—C12	1.332 (2)	C12—C13	1.390 (2)
N4—C16	1.346 (2)	C12—H12	0.9500
N4—Cd1^iv^	2.3728 (14)	C13—C14	1.380 (3)
C1—C2	1.386 (2)	C14—C15	1.390 (3)
C1—H1	0.9500	C14—H14	0.9500
C2—C3	1.389 (3)	C15—C16	1.376 (3)
C2—C6	1.508 (2)	C15—H15	0.9500
C3—C4	1.381 (3)	C16—H16	0.9500
C3—H3	0.9500		
			
N4^i^—Cd1—N4^ii^	180.0	C2—C6—H6B	109.2
N4^i^—Cd1—N1	94.21 (5)	H6A—C6—H6B	107.9
N4^ii^—Cd1—N1	85.79 (5)	N2—C7—C8	110.71 (15)
N4^i^—Cd1—N1^iii^	85.79 (5)	N2—C7—H7A	109.5
N4^ii^—Cd1—N1^iii^	94.21 (5)	C8—C7—H7A	109.5
N1—Cd1—N1^iii^	180.00 (5)	N2—C7—H7B	109.5
N4^i^—Cd1—Cl1	90.60 (4)	C8—C7—H7B	109.5
N4^ii^—Cd1—Cl1	89.40 (4)	H7A—C7—H7B	108.1
N1—Cd1—Cl1	87.57 (4)	N3—C8—C7	109.98 (16)
N1^iii^—Cd1—Cl1	92.43 (4)	N3—C8—H8A	109.7
N4^i^—Cd1—Cl1^iii^	89.40 (4)	C7—C8—H8A	109.7
N4^ii^—Cd1—Cl1^iii^	90.60 (4)	N3—C8—H8B	109.7
N1—Cd1—Cl1^iii^	92.43 (4)	C7—C8—H8B	109.7
N1^iii^—Cd1—Cl1^iii^	87.57 (4)	H8A—C8—H8B	108.2
Cl1—Cd1—Cl1^iii^	180.0	N3—C9—C10	110.96 (15)
H1WA—O1W—H1WB	100 (2)	N3—C9—H9A	109.4
H2WA—O2W—H2WB	100 (2)	C10—C9—H9A	109.4
C1—N1—C5	117.70 (16)	N3—C9—H9B	109.4
C1—N1—Cd1	116.82 (11)	C10—C9—H9B	109.4
C5—N1—Cd1	124.63 (12)	H9A—C9—H9B	108.0
C7—N2—C6	111.94 (14)	N2—C10—C9	110.80 (15)
C7—N2—C10	109.05 (14)	N2—C10—H10A	109.5
C6—N2—C10	108.82 (14)	C9—C10—H10A	109.5
C8—N3—C9	109.92 (14)	N2—C10—H10B	109.5
C8—N3—C11	113.01 (15)	C9—C10—H10B	109.5
C9—N3—C11	111.90 (15)	H10A—C10—H10B	108.1
C12—N4—C16	117.41 (15)	N3—C11—C13	109.80 (14)
C12—N4—Cd1^iv^	119.03 (11)	N3—C11—H11A	109.7
C16—N4—Cd1^iv^	123.53 (11)	C13—C11—H11A	109.7
N1—C1—C2	123.82 (16)	N3—C11—H11B	109.7
N1—C1—H1	118.1	C13—C11—H11B	109.7
C2—C1—H1	118.1	H11A—C11—H11B	108.2
C1—C2—C3	117.32 (16)	N4—C12—C13	124.16 (16)
C1—C2—C6	120.65 (16)	N4—C12—H12	117.9
C3—C2—C6	122.03 (16)	C13—C12—H12	117.9
C4—C3—C2	119.52 (17)	C14—C13—C12	117.45 (16)
C4—C3—H3	120.2	C14—C13—C11	125.13 (16)
C2—C3—H3	120.2	C12—C13—C11	117.42 (16)
C5—C4—C3	118.99 (18)	C13—C14—C15	119.35 (16)
C5—C4—H4	120.5	C13—C14—H14	120.3
C3—C4—H4	120.5	C15—C14—H14	120.3
N1—C5—C4	122.63 (18)	C16—C15—C14	118.91 (17)
N1—C5—H5	118.7	C16—C15—H15	120.5
C4—C5—H5	118.7	C14—C15—H15	120.5
N2—C6—C2	112.20 (14)	N4—C16—C15	122.71 (16)
N2—C6—H6A	109.2	N4—C16—H16	118.6
C2—C6—H6A	109.2	C15—C16—H16	118.6
N2—C6—H6B	109.2		
			
N4^i^—Cd1—N1—C1	138.25 (13)	C10—N2—C7—C8	−57.93 (19)
N4^ii^—Cd1—N1—C1	−41.75 (13)	C9—N3—C8—C7	−59.1 (2)
Cl1—Cd1—N1—C1	−131.32 (12)	C11—N3—C8—C7	175.07 (15)
Cl1^iii^—Cd1—N1—C1	48.68 (12)	N2—C7—C8—N3	60.0 (2)
N4^i^—Cd1—N1—C5	−52.61 (14)	C8—N3—C9—C10	58.00 (19)
N4^ii^—Cd1—N1—C5	127.39 (14)	C11—N3—C9—C10	−175.57 (14)
Cl1—Cd1—N1—C5	37.82 (14)	C7—N2—C10—C9	56.28 (19)
Cl1^iii^—Cd1—N1—C5	−142.18 (14)	C6—N2—C10—C9	178.64 (15)
C5—N1—C1—C2	−0.8 (3)	N3—C9—C10—N2	−57.0 (2)
Cd1—N1—C1—C2	169.09 (13)	C8—N3—C11—C13	−153.02 (16)
N1—C1—C2—C3	1.8 (3)	C9—N3—C11—C13	82.25 (18)
N1—C1—C2—C6	−177.44 (16)	C16—N4—C12—C13	0.1 (3)
C1—C2—C3—C4	−1.5 (3)	Cd1^iv^—N4—C12—C13	−178.06 (13)
C6—C2—C3—C4	177.80 (17)	N4—C12—C13—C14	0.7 (3)
C2—C3—C4—C5	0.2 (3)	N4—C12—C13—C11	−179.79 (16)
C1—N1—C5—C4	−0.6 (3)	N3—C11—C13—C14	−129.54 (18)
Cd1—N1—C5—C4	−169.62 (14)	N3—C11—C13—C12	51.0 (2)
C3—C4—C5—N1	0.9 (3)	C12—C13—C14—C15	−1.0 (3)
C7—N2—C6—C2	−60.88 (19)	C11—C13—C14—C15	179.54 (18)
C10—N2—C6—C2	178.52 (15)	C13—C14—C15—C16	0.5 (3)
C1—C2—C6—N2	112.33 (18)	C12—N4—C16—C15	−0.7 (3)
C3—C2—C6—N2	−66.9 (2)	Cd1^iv^—N4—C16—C15	177.39 (14)
C6—N2—C7—C8	−178.39 (15)	C14—C15—C16—N4	0.4 (3)

Symmetry codes: (i) *x*+1/2, −*y*+3/2, *z*+1/2; (ii) −*x*−1/2, *y*−1/2, −*z*+1/2; (iii) −*x*, −*y*+1, −*z*+1; (iv) −*x*−1/2, *y*+1/2, −*z*+1/2.

### Hydrogen-bond geometry (Å, °)

**Table:**

*D*—H···*A*	*D*—H	H···*A*	*D*···*A*	*D*—H···*A*
O1W—H1WA···N2	0.87 (2)	2.08 (2)	2.950 (2)	171 (2)
O1W—H1WB···O2W^v^	0.88 (2)	2.01 (2)	2.838 (3)	156 (2)
O2W—H2WA···O1W	0.92 (2)	1.96 (2)	2.844 (3)	161 (3)
O2W—H2WB···Cl1^vi^	0.91 (2)	2.27 (2)	3.1607 (17)	165 (3)

Symmetry codes: (v) −*x*+1, −*y*+2, −*z*+1; (vi) −*x*+1/2, *y*+1/2, −*z*+3/2.
